# Supplementary material for: Modular Design of Programmable Mechanofluorescent DNA Hydrogels
Source: Nat Commun. 2019 Jan 31;10:528. doi: 10.1038/s41467-019-08428-2 (PMC6355893; doi:10.1038/s41467-019-08428-2)
Supplement: Supplementary file 1 — Supplementary Information [file 41467_2019_8428_MOESM1_ESM.pdf]

Supplementary Information for:

Modular Design of Programmable Mechanofluorescent DNA Hydrogels

Walther *et al.*

Supplementary Methods

Materials. ssDNA oligomers were purchased from Integrated DNA Technologies (IDT) as summarized in Supplementary Table 1. The enzymes T_4 ligase (low concentration 2 $U{\cdot}\mu L^{-1}$ and high concentration 20 $U{\cdot}\mu L^{-1}$), Exonuclease I (40 $U{\cdot}\mu L^{-1}$), Exonuclease III (200 $U{\cdot}\mu L^{-1}$), Inorganic pyrophosphatase (2 $U{\cdot}\mu L^{-1}$) and Φ_{29} polymerase (10 $U{\cdot}\mu L^{-1}$) were purchased from Lucigen. Deoxynucleotide triphosphate (dATP, dTTP, dGTP and dCTP) 100 µM 1 mL and dUTP-Cy5 1 mM 10 µL were purchased from Jena bioscience. Magnesium acetate tetrahydrate ($MgAc_2$), Tween 80, tris(hydroxymethyl)aminomethane hydrochloride (TRIS-HCl and Trizma buffer substance pH=8), disodium ethylenediaminetetraacetate dehydrate (EDTA) and acetic acid were purchased (as bioreagent grade if available) from Sigma-Aldrich. Agarose Low EEO was purchased from AppliChem. $SYBR_{Gold}$ and 50 bp and 1 kbp ladders were purchased from Thermo Fisher Scientific. Unless stated otherwise, the base of all solutions is a TE buffer that consists of 10 mM of Tris(hydroxymethyl)aminomethane (pH=8.0) and 1 mM of EDTA, for module folding and hydrogel self-assembly the TE also contain 100 mM of NaCl and 12 mM of $MgAc_2$. DNA sequences are also stored frozen at -25 °C in TE buffer without extra salt. Gel electrophoresis (GEP) were run using 2 wt% agarose gels in TAE buffer containing 40 mM of TRIS-HCl, 20 mM of acetic acid and 1 mM of EDTA. $SYBR_{Gold}$ staining was performed after GEP by placing the agarose gel in 40 mL of running buffer containing 1x $SYBR_{Gold}$ for 10 min.

Supplementary Table 1. DNA sequences used, with their name, the sequence code used for ordering at IDT, the purification grade and modifications.

	Name	Sequence 5'→3'	Purification	Modification
Templates for RCA[a]	$A_4*A_3*X*A_2*A_1*$	/5phos/GTA GGT GGA CAC GCC GCA TCC TAC ATC CAG CGT CAA CGT CGG ACG CGA TTC ATT GAC CAG TCA GCC GCC ACC	HPLC	5'-Phosphorylation
	$B_4*B_3*XB_2*B_1*$	/5phos/GAG CTG CCG CGC GAG CAA TAA CGG TAC AGC CCG ACG TTG ACG GAT ACG CTC AAC AGG CAG CGG GCG CTT CGC	HPLC	5'- Phosphorylation
Ligation strands	A'_1A_4	CCA CCT ACG GTG GCG GCT GA	HPLC	None
	B'_1B_4	GGC AGC TCG CGA AGC GCC CG	HPLC	None
Primers for RCA	X	CCG ACG TTG A*C*G	Desalting	Phosphorothioated Twice
	X*	CGT CAA CGT C*G*G	Desalting	Phosphorothioated Twice
Functionalization	$Atto_{488}$-A_2*	/5ATTO488N/ACG CGA TTC ATT GAC	HPLC	5' Atto 488 (NHS ester)
	$Atto_{565}$-MA_4*	/5ATTO565N/CGC GTT GCG CCT GCC GTA GGT GGA CAC GCC	HPLC	5' Atto 565 (NHS ester)
	B_4*N-IowaRQ	CAG CGG GCG CTT CGC GAG CCG CGC ACG CCG/3IAbRQSp/	HPLC	5' Iowa Black® RQ
	B_4*N	CAG CGG GCG CTT CGC GAG CCG CGC ACG CCG	Desalting	None
	MA_4*	CGC GTT GCG CCT GCC GTA GGT GGA CAC GCC	Desalting	None
	A_1*M*D_1*	CAG TCA GCC GCC ACC GGC AGG CGC AAC GCG CGG CCG CGC GCC CGG	Desalting	None
	D_1N*B_1*	CCG GGC GCG CGG CCG CGG CGT GCG CGG CTC GAG CTG CCG CGC GAG	Desalting	None
	A_1*M*D_1*	CAG TCA GCC GCC ACC GGC AGG CGC AAC GCG CGG CCG CGC GCC CGG	PAGE	None
	D_1N*B_1*	CCG GGC GCG CGG CCG CGG CGT GCG CGG CTC GAG CTG CCG CGC GAG	PAGE	None
	A_1*M*D_2*	CAG TCA GCC GCC ACC GGC AGG CGC AAC GCG CGT CCG AC	PAGE	None
	D_2N*B_1*	GTC GGA CG CGG CGT GCG CGG CTC GAG CTG CCG CGC GAG	PAGE	None
	$A_1*M*HPN*B_1*$	CAG TCA GCC GCC ACC GGC AGG CGC AAC GCG CGT CCG ACT TTT TTG TCG GAC GCG GCG TGC GCG GCT CGA GCT GCC GCG CGA G	PAGE	None
	$A_1*M*TN*B_1*$	CAG TCA GCC GCC ACC GGC AGG CGC AAC GCG TTT TTT CGG CGT GCG CGG CTC GAG CTG CCG CGC GAG	PAGE	None

[a]These sequences are converted into polymers using RCA. Hence, the complementary nucleobase base structure is found in the multiblock copolymers.

Instrumentation. Thermal ramps were performed on a Personal Thermocycler (Analytik Jena), DNA concentrations were measured using a ScanDrop (Analytik Jena) spectrophotometer with a standard value of 33 µg per OD_{260} for ssDNA. Confocal laser scanning microscopy (CLSM) was performed on Leica TCS SP8 microscope. Widefield fluorescent imaging was performed using a fluorescent AxioZoom (Zeiss) Microscope. Rheology experiments were performed on a MCR 301 Rheometer from Anton Paar with a 15 mm plate/plate geometry set up. Typically, 45 µL of a given solution were loaded in the rheometer, and the plate/plate distance was set to 0.2 mm. A drop of mineral oil (Sigma-Aldrich) was applied on the periphery of the plate to prevent water evaporation during the temperature ramp. Except when stated otherwise the rheology measurements are performed at constant amplitude (5 %) and constant frequency (1 Hz).

Supplementary Note 1: Hydrogel Synthesis

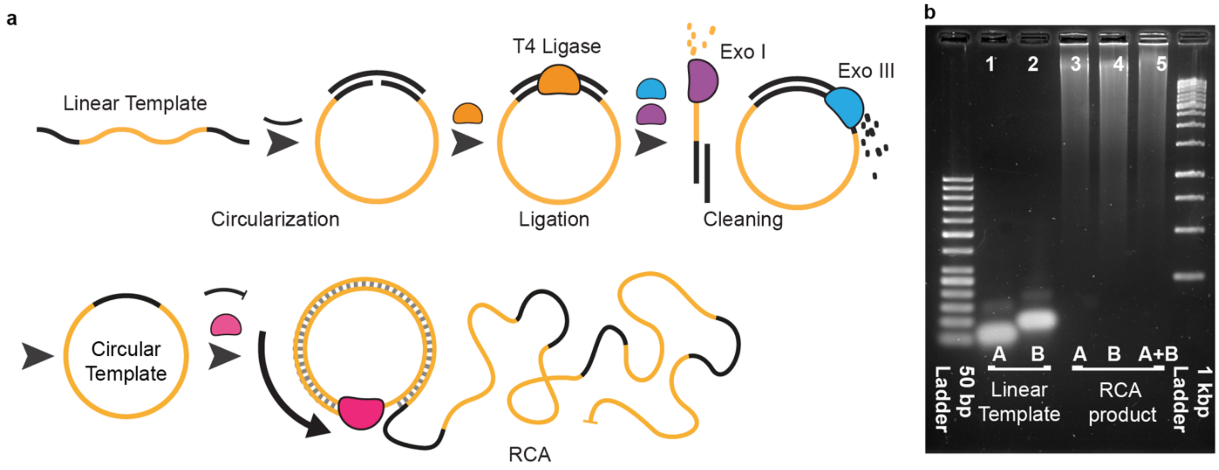

Supplementary Figure 1: Synthesis of the hydrogel precursor via Rolling Circle Amplification (RCA). (a) Scheme of the entire process and (b) gel electrophoresis (GEP) of the short linear templates (Lanes 1 and 2) and the corresponding amplified hydrogel precursors (lanes 3 and 4). Mixing of hydrogel precursors A and B at room temperature and low concentration results in a liquid ssDNA solution that migrates in the GEP. The GEP were run in 2 wt% agarose, TAE buffer for 90 min at 90 V and post-stained with $SYBR_{Gold}$. The 50 bp ladder bands correspond to 50, 100, 150, 200, 250, 300, 400, 500, 600, 700, 800, 900 and 1000 base pairs; the 1kbp ladder bands correspond to 250, 500, 750, 1000, 1500, 2000, 2500, 3000, 3500, 4000, 5000, 6000, 8000 and 10000 bp from top to bottom.

Supplementary Note 2: Impact of module functionalization on rheological properties

We report the impact of strain and frequency on the measured rheological properties of the DNA hydrogel before and after functionalization with module D_1 (Supplementary Figure 2a,b). The hydrogel functionalization was analyzed in-situ by first analyzing the pregel and then functionalization of it with the D_1 module in the rheometer.

First, 40 µL of 0.7 wt% pregel (in TE buffer containing 12 mM of $MgAc_2$ and 100 mM of NaCl) were loaded into the rheometer and subsequently set into a solid gel by running a temperature ramp from 10 to 80 and to 10 °C with 5 min stay at 80 °C and at 3 $°C \cdot min^{-1}$ heating and cooling rate. Subsequently, we analyzed this unfunctionalized gel more deeply, by strain sweep experiments from 0.1 to 300% at 0.1 Hz (Supplementary Figure 2a) and via a frequency sweep from 0.01 up to 100 Hz at 1% strain (Supplementary Figure 2b). To allow for further functionalization with the D_1 module, we removed any strain or stress-dependent properties in the hydrogel by re-annealing using a temperature ramp (heating to 80 °C then cooling back to 10 °C) while recording the variation of the mechanical properties with temperature upon cooling (Supplementary Figure 2c).

After removing the rheometer upper plate, we loaded 345 µL D_1 module (20 µM, equimolar to the functionalization sites A_1, A_4, B_1 and B_4) in TE buffer containing 12 mM of $MgAc_2$ and 100 mM of NaCl onto the hydrogel, and left the module to diffuse and hybridize into the DNA hydrogel overnight at room temperature. After ca. 10 h, we placed back the upper rheometer plate. Since fluorescent and PAGE-purified strands are too expensive to run experiments at such scale, we used non-fluorescent strands with standard purification to assemble the modules used for rheology characterization. Hybridization of the modules in the DNA hydrogel induces an increase of the volume of about 50%, as seen by the gap distance increase of the rheometer plates from 0.2 mm to 0.3 mm to adapt to the new thickness. We then ran similar experiments as above for the non-functional gel, i.e. a strain sweep from 0.1 to 300% strain at 0.1 Hz (Supplementary Figure 2a), as well as a new frequency sweep from 0.01 up to 100 Hz at 1% strain (Supplementary Figure 2b). Finally, we performed the same temperature ramp as before (to remove strain or stress-induced damages) while recording variations of modulus upon cooling (Supplementary Figure 2c).

Initially surprising, the loss and storage modulus of the hydrogels remained very similar before and after functionalization with module D_1. However, it needs to be considered that the volume increase by 50% (strength decrease) compensates the increase of crosslink density (strength increase) associated with the hybridization of the module inside the hydrogel.

Strain sweeps show an earlier decrease of storage modulus for D_1-functionalized hydrogels compared to the pristine DNA hydrogel. Here, the swelling of the gel associated with module hybridization stretches the initial network and results in network failure at lower strain. This decrease of storage modulus corresponds to an increase of loss modulus because strain-induced reorganization of the network of DNA bonds (sacrificial bonds) allows energy dissipation. A similar yet less pronounced increase of loss modulus upon failure is also visible for the pristine DNA hydrogel. The frequency sweeps are nearly identical before and after functionalization indicating that crosslinking with module D_1 does not change the network temporal response, while again, one has to bear in mind that the hydrogel is diluted to 50 % and has an increased amount of crosslinks in total. Finally, the most dramatic differences associated to the hybridization of D_1 module are visible during temperature ramps, as displayed in the cooling curves after removing strain/stress-induced damage (Supplementary Figure 2c). The pristine DNA hydrogel shows a single increase of loss and storage modulus near 70 °C, corresponding to X/X* hybridization. However, after functionalization, multiple transitions for the loss modulus are observed, which indicates a rich variety of transient supramolecular bond formation across the network. These new transient networks further induce an increase of storage modulus between 45 and 65 °C compared to the pristine hydrogel. While, this peculiar response is relevant and reproducible, we suggest that the in-depth evaluation of these phenomena is subject to further rheological characterization beyond the conceptual scope of this manuscript to clearly identify and explain these variations on a consistent structural basis.

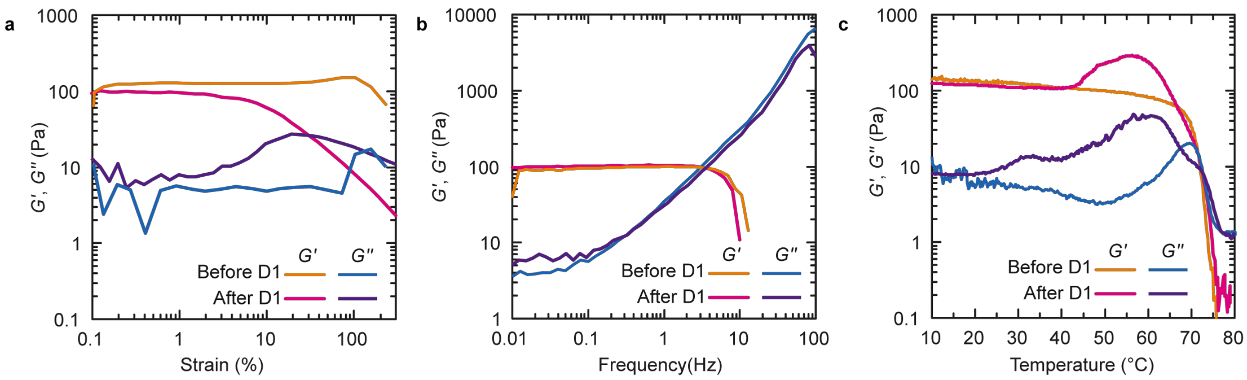

Supplementary Figure 2: Rheological properties of pristine and D_1 functionalized DNA hydrogels (a) Amplitude sweep of pristine 0.7 wt% DNA hydrogel and DNA hydrogel functionalized with D_1 module at a frequency of 0.1 Hz. (b) Frequency sweep of pristine 0.7 wt% DNA hydrogel and DNA hydrogels functionalized with D_1 module at 1% strain. (c) Temperature dependent (cooling ramp) modulus of pristine 0.7 wt% DNA hydrogel and DNA hydrogel functionalized with module D_1, performed at a frequency of 0.1 Hz and 1% strain.

Supplementary Note 3: Sample preparation for mechanical testing

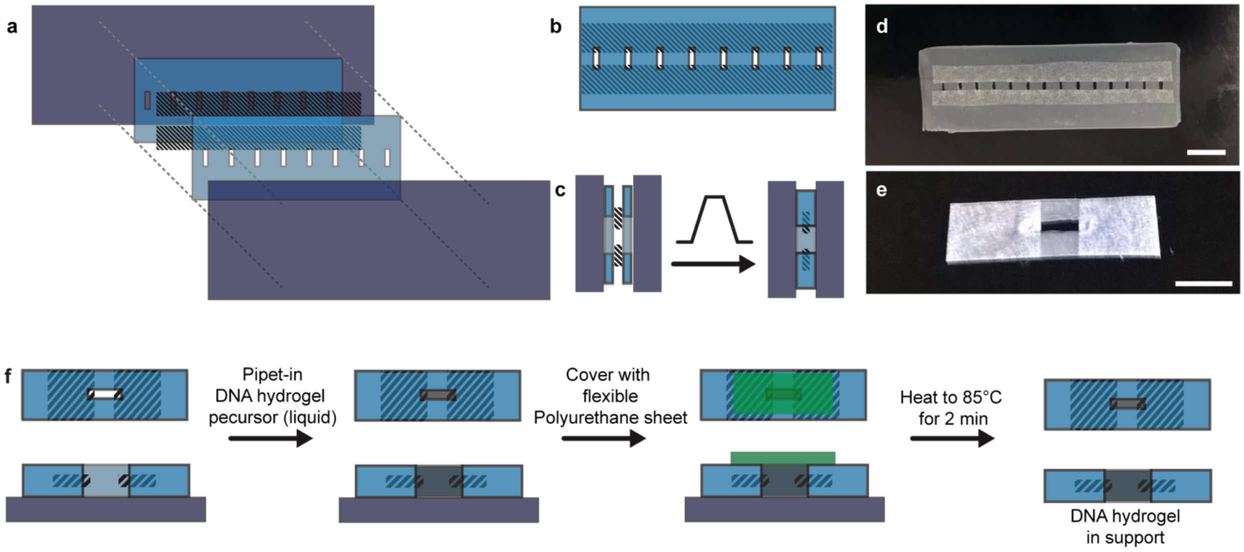

Supplementary Figure 3: Sample holder fabrication and preparation of suspended hydrogels. (a) 3D exploded view of the sandwich structure composed of 2 strips of dust-free Kimtech Precision Whipe paper (dashed grey), sandwiched between two pre-cut Parafilm sheets (light blue), and placed between 2 glass slides. (b) Top view of the sandwich and (c) side view before and after heat treatment which melts the Parafilm and traps the paper strips leaving a few mm overhang that will hold the DNA hydrogel. (d, e) Photograph of the entire sandwich after thermal mending with (d) 14 individual sample holder (scale bar = 1 cm) and (e) a single sample holder after cutting (scale bar = 0.5 cm). (f) Scheme of the filling of the sample holder, sealing of it with a soft polyurethane sheet, and heat-induced crosslinking of the DNA hydrogel.

Supplementary Note 4: Characterization of force-sensing DNA modules

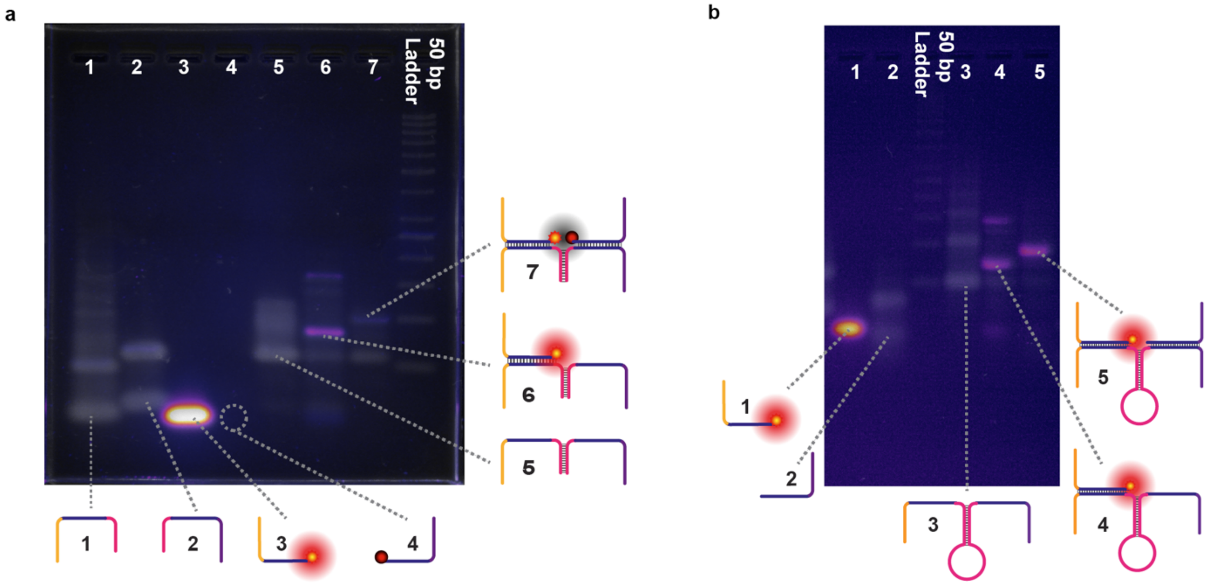

Supplementary Figure 4: Folding of the mechanosensing units. Bicolor GEP of various partial or complete mechanosensing modules. The gels are imaged first before $SYBR_{gold}$ staining where only fluorescent constructs are visible (Blue-red-yellow color scale:) and after $SYBR_{gold}$ staining (black and white Scale). (a) GEP of the module D_1 components. Lane 1: A_1*M*D_1*, lane 2: D_1N*B_4*, lane 3: $Atto_{565}$-MA_4*, lane 4: B_1*N-IowaRQ (invisible because of the quencher), lane 5: A_1*M*D_1* + D_1N*B_4*, lane 6: A_1*M*D_1* + D_1N*B_4*+ A_1*M*D_1* + D_1N*B_4*, lane 7: A_1*M*D_1* + D_1N*B_4*+ A_1*M*D_1* + D_1N*B_4*+ B_1*N-IowaRQ (full module D_1 poorly visible because of the quencher). (b) GEP of the components of module HP. Lane 1: $Atto_{565}$-MA_4*, lane 2: B_1*N (without quencher for visibility), lane 3: A_1*M*HPN*B_4*, lane 4: A_1*M*HPN*B_4* + $Atto_{565}$-MA_4*, lane 5: A_1*M*HPN*B_4* + $Atto_{565}$-MA_4* + B_1*N (Full module HP without quencher for visibility). The GEP were run in 2 wt% agarose, TAE buffer for 90 min at 90 V. The 50 bp ladder bands correspond to 50, 100, 150, 200, 250, 300, 400, 500, 600, 700, 800, 900 and 1000 bp.

Supplementary Note 5: Melting transitions and fluorescence responses of the mechanosensing modules

We measured the melting transitions of the different modules by temperature-dependent fluorescence spectroscopy. The modules were assembled according to the previous protocol and diluted to 1 µM in TE buffer containing 12 mM of $MgAc_2$ and 100 mM of NaCl. Subsequently, 50 µL were introduced in a 3 x 3 mm fluorescence microcuvette (Hellma Analytics) and covered with a drop of oil to prevent evaporation. We recorded the fluorescence using an Ocean Optics spectrometer equipped with a Peltier-heated cuvette holder. An exemplary heating profile for the D_1 modules is presented Supplementary Figure 5a and consists of two symmetric heating/cooling cycles between 15 and 85 °C at 2 °C·min^{-1} with 2 min equilibration time at 85 °C and 15 °C. The fluorescence spectrums were recorded using 490 nm as excitation wavelength. The maximum of emission at 600 nm is plotted as a function of time for module D_1 for two heating cycles (Supplementary Figure 5a). Supplementary Figure 5b compares the different modules by plotting the fluorescence response as a function of temperature (2nd heating ramp). The same increase of fluorescence is observed around 70 °C for modules D_1, HP and T, while the module D_2 melts around 45 °C. The similar response for D_1, HP and T relates to the meltings of the M (79 °C) or N (77 °C) arms which are predicted to happen at slightly lower temperature than D_1 (84 °C) or HP (78 °C). In stark contrast, in D_2, the breakage of the sacrificial bonds is clearly observed, corresponding to the expected melting transition (50 °C in Table 2, main MS). Note that the 5 °C discrepancy between observed and calculated values arises from the difference of concentrations used for T_m simulation (10 µM DNA) and for fluorescence measurements (1 µM DNA). Values at 10 µM are more realistic concerning the material and rheological behavior.

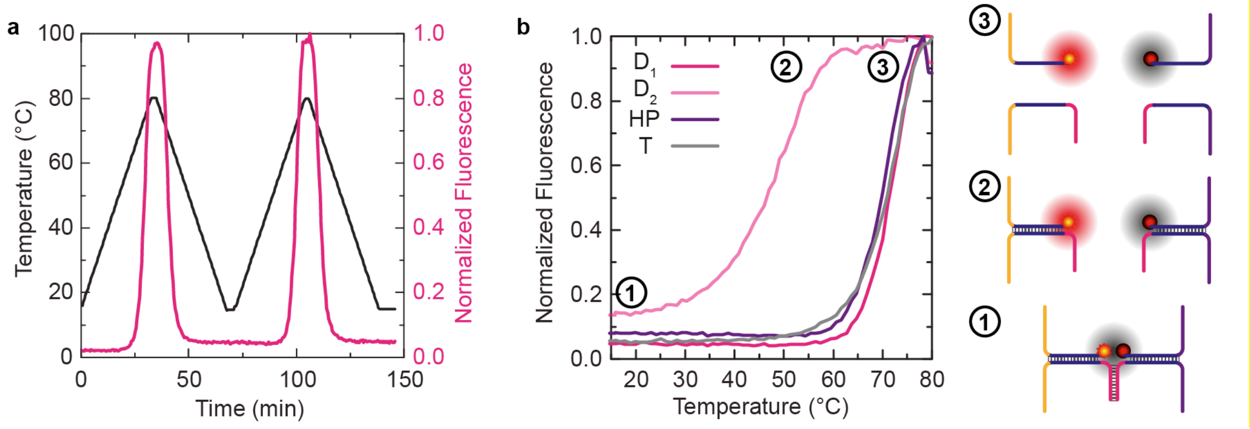

Supplementary Figure 5: Fluorescence monitoring of the melting transition of the mechanosensing modules. (a) Temperature profile (black line) and the corresponding fluorescence at 600 nm of module D_1. The fluorescence is normalized to its maximum intensity observed at 85 °C. (b) Comparison of normalized fluorescence at 600 nm for the modules D_1, D_2, HP and T as function of the temperature together with schematic representation of the disassembly state at various temperatures. All curves are recorded at a module concentration of 1 µM in TE buffer containing 12 mM of $MgAc_2$ and 100 mM of NaCl.

Supplementary Note 6: Mechanofluorescence characterization

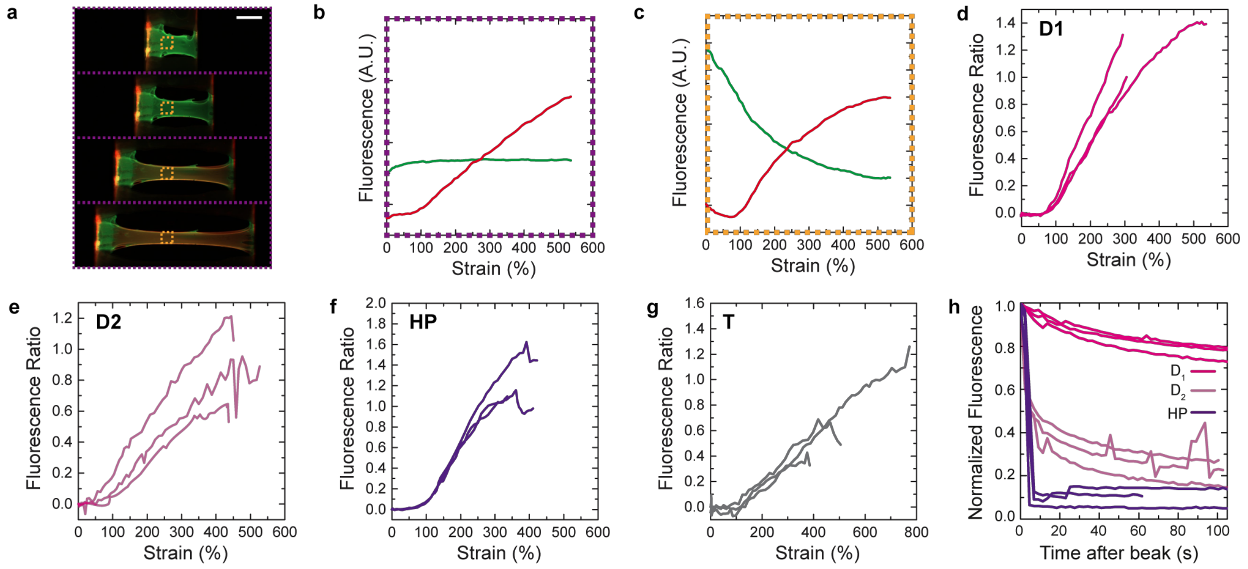

Supplementary Figure 6: Mechanofluorescence characterization. (a) Example of a series of fluorescence images at different stretching ratios of a 0.7 wt% hydrogel functionalized with the mechanosensory module D_1 (scale bar = 1 mm) (b) Evolution of the average red and green fluorescence over the entire image (purple square) upon stretching. (c) Evolution of the average red and green fluorescence inside the yellow square upon stretching. (d-g) Superposition of three individual measurement performed for each module D_1 (d), D_2 (e), HP (f) and T (g). (h) Superposition of three individual temporal recovery experiment for each module D_1, D_2 and HP.

Supplementary Note 7: Temporal recovery experiments

Figure 3d of the main manuscript displays different mechanofluorescent modules that lead to different temporal recovery behavior. The mechanism for irreversible fluorescence recovery is presented in Supplementary Figure 7a. For the two modules with a simple sacrificial duplex (D_1 and D_2) there is a competition between self-dimer formation and reformation of the original hetero-duplex. Since each side of the mechanofluorescent module binds to one of the RCA products they remain in close proximity after cleavage of the sacrificial duplex which gives a kinetic advantage to self-dimer (D_x/D_x and D_x*/D_x*, x = 1, 2) formation, while the heterodimer (D_x/D_x*, x = 1, 2) is thermodynamically favored because it maximizes the number of base pairs. The difference between module D_1 and D_2 lies in the stability of the self-dimers. NUPACK (http://www.nupack.org) simulations show that the self-dimers of D_1 are stable at RT (25 °C; Supplementary Figure 7c-d) while the self-dimers of D_2 are unstable at RT (Supplementary Figure 7f-g). The hetero-complimentary dimers of D_1/D_1* and D_2/D_2* are all stable at RT (Supplementary Figure 7b,e). In D_1 the formation and relative stability of the kinetic products slows down the reformation of the original FRET pair and fluorescence decrease after stress release. On contrary, for module D_2 the lack of stability of the self-dimers at RT yields a spontaneous recovery of the more stable original duplex D_2/D_2*. This recovery is nevertheless slow because the two arms need to find each other by diffusion while remaining attached to the hydrogel network. For the last one, HP, the two arms of the module are covalently linked, and they cannot move far apart. The module HP therefore quickly returns to its original conformation after stress release, as it is both kinetically and thermodynamically favored (Supplementary Figure 7h). This results macroscopically in a fast fluorescence decrease.

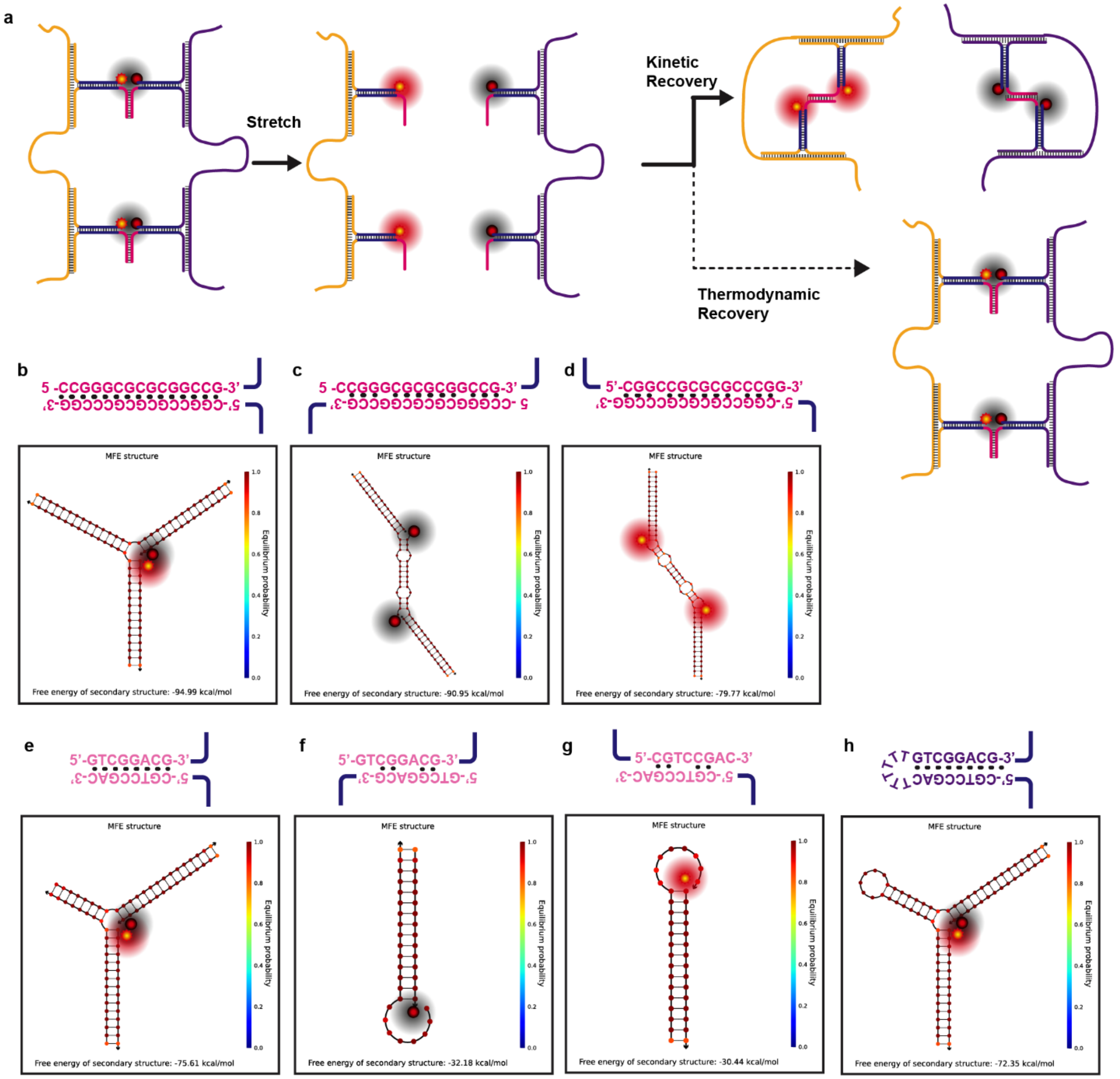

Supplementary Figure 7: Control over kinetic and thermodynamic recovery leading to reversible and irreversible mechanofluorescence. (a) Schematic representation of the kinetic and thermodynamic recovery pathways, where self-binding happens faster due to the proximity of similar halves of the modules in the hydrogel matrix (the fluorophore side of the module binds to RCA product A, while the quencher side binds to the RCA product B). Reformation of the original modules yields more stable duplexes. (b-d) The most stable structure at 25 °C according to NUPACK for module D_1 shows that even though the initial D_1/D_1^* duplex of the perfectly folded module is most stable (D_1/D_1^*; in b), both D_1/D_1 (in c) and D_1^*/D_1^* (in d) can form stable self-duplexes. (e-g) On the contrary, NUPACK simulations for module D_2 show that only the original D_2/D_2^* can form stable structures at 25 °C (in e), while the D_2/D_2 (in f) and D_2^*/D_2^*(in g) remain single stranded in the absence of their counterpart as seen by the non-bound loop area at the bottom and top, respectively. (h) The HP duplex forms a stable structure at 25 °C according to NUPACK. Note that in this case this is also the kinetic product as both ends of the duplex are connected. All simulations represent the structure of the most probable product with 10 µM of DNA strand at 25 °C. To probe the stability of the kinetic products in (c, d, f, g) only one half of the sacrificial sensory duplex was considered for the simulation.
